# Shared Destiny in the Digital Era: Sensation Seeking, Loneliness, and Excessive Internet Use among Middle-Aged and Older Adults in Mainland China

**DOI:** 10.3390/ijerph192113766

**Published:** 2022-10-23

**Authors:** Heng Yang, Jianbin Jin

**Affiliations:** 1 School of Journalism and Communication, Tsinghua University, Beijing 100084, China; 2 Department of Communication and Media Research, University of Zurich, 8050 Zurich, Switzerland

**Keywords:** excessive internet use, sensation seeking, loneliness, digital leisure, well-being, successful aging

## Abstract

With digital infrastructures becoming the foundation of modern life and a shared lifestyle, the internet has become a popular leisure tool for middle-aged and elderly individuals. However, inappropriate use of the internet can jeopardize their health and quality of life, and excessive internet use by middle-aged and older adults is a cause for concern. This study found that middle-aged and older adults are vulnerable to excessive internet use. One predictor of excessive use is loneliness, but its effect is relatively limited. It is a mediating variable rather than the essential cause of excessive internet use by middle-aged and older adults. The effect of sensation seeking is a strong predictor of middle-aged and older adults’ excessive internet use, which means they have a strong desire to use the internet to satisfy their emotional needs, thus, resulting in excessive internet use. The social nature of digital infrastructure in a relational framework and the impact of the internet on different populations are likely more complex than we imagine and have the potential to cause many unintended effects.

## 1. Introduction

The simultaneous development of an aging world population and the globalization of information and communication technology (ICT) has made the use of the internet, especially its excessive use, by the elderly a social phenomenon and academic issue of concern. The number of adults over 65 will grow from approximately 1 billion in 2030 to 1.6 billion by 2090, and the number of people over the age of 100 will increase tenfold by 2050 [1]. Compared to the global rate of aging, China’s aging society is growing at an alarming rate. The country’s elderly population reached 241 million in 2017, accounting for 17.3% of the Chinese population, and this share is expected to continue to climb to more than 35% by 2050 [2].

Along with aging comes the deep penetration of ICT into society. As one of the common digital infrastructures, the internet has become a foundation of modern life for young and old alike. The exponential growth in the use of the internet among middle-aged and older adults around the world is well established. In the EU, 42% of seniors aged 64–74 use the internet daily, with digital media use becoming one of their regular daily activities; in the US, 73% of seniors over the age of 65 use the internet [3]. In China, the number of internet users over the age of 50 has reached a staggering 277 million [4].

Studies have found that age is a key factor associated with ICT use and digital equality [5]. The digital media use of older adults is seen as an important issue related to social inclusion and social justice [6]. The adoption and proper use of digital media is currently understood as an essential aspect of “successful aging” [7]. Therefore, issues related to the digital divide and the digital inclusion of older adults have received increasing attention from researchers in recent years. In our perception, age tends to be associated with reduced health and resources, as well as diminished openness to new experiences [8]. Driven by this stereotype, most studies have been conducted from the perspectives of innovation diffusion, technology adoption, or uses and gratifications, focusing on the adoption status, usage patterns, facilitators, and barriers to adoption of the internet among middle-aged and older adults [9,10]. Little consideration has been given to the individual and social consequences that internet use has for middle-aged and older adults and the relationship between their internet usage and their life status. The risks and negative effects of the internet for middle-aged and older adults have been neglected. To some extent, this is a reflection of “pro-innovation bias” in academic research as well.

The rapid globalization of the internet has also caused globalization of its negative effects and created new types of problems that were previously nonexistent. Now, it is a very common phenomenon for Chinese middle-aged adults and elders to use the internet for socializing and entertainment, and they show obvious internet withdrawal reactions, such as anxiety and restlessness, when they cannot access the internet or spend less time on the internet, which are very similar to the criteria of internet addiction proposed by Young (2004). In addition, with the persistence and recurrence of the COVID-19 pandemic in China in recent years, the use of the internet by middle-aged and elderly individuals has further accelerated [11]. According to official Chinese statistics, compared to the pre-pandemic period (August 2019), the proportion of older internet users aged 50+ in China jumped rapidly from 13.6% to 26.8% in February 2022, with the population growing from 99 million to 277 million [4]. On the one hand, the government monitors and tracks the public’s health and travel status through various methods, such as health QR codes and travel QR codes, and people need to learn to use such apps to cross this artificial digital barrier to live and travel relatively normally; this practice objectively promotes the use of the internet among middle-aged and elderly individuals. On the other hand, the frequent pandemic lockdown measures across China have greatly limited the opportunities for middle-aged and elderly individuals to leave their homes, and it has become a common practice for them to spend their days at home online. Going digital has become a necessity for middle-aged and older adults. Research has found that older adults rely more on the internet since the pandemic began, and this trend could be part of the “new normal”, even after COVID-19, as older adults adapt to a more mobile-app-dependent lifestyle [12]. This trend has widespread social effects. In China, some middle-aged and older people have had their lives disrupted by internet use, have neglected the care and companionship of family members, and are more vulnerable to online telecommunications fraud. The overuse of the internet by middle-aged and elderly individuals is even showing signs of becoming a social as well as a public health problem in China.

## 2. Literature Review

### 2.1. Leisure, (Excessive) Internet Use, Middle-Aged and Elderly Individuals

Leisure activity refers to the use of discretionary time to do things for the enjoyment of the actor and is usually performed by choice [13]. It may evoke good feelings in people when they engage in it, and the interplay between leisure and well-being has been variously expressed as life satisfaction, happiness, quality of life, and wellness [14]. It has been viewed as an antidote to alienated labor and as a route towards a well-lived life and plays an important role in the health, well-being, and quality of life of individuals [15]. It has been found that older adults’ participation in leisure activities is associated with lower loneliness and positive well-being [16,17]. When leisure needs are not satisfied, the subjective well-being of middle-aged and older adults is undermined, for example, by a decline in job satisfaction [18]. In gerontology, well-being is said to be the subjective counterpart of a more public evaluation of ‘successful aging’ [19]. Therefore, enhancing the well-being of middle-aged and older adults through participation in leisure activities is seen as an important aspect of successful aging.

Currently, digital devices based on ICT enable individuals to develop what we may call digital leisure activities, and, for many people, they have replaced leisure activities in reality [20]. The same is happening for middle-aged and elderly individuals, who are also increasingly involved in the digital leisure activities represented by the internet, and, therefore, the boundaries between real and digital leisure activities are diminishing for them [21]. Digital leisure activities can bring similar satisfaction to traditional leisure activities, such as spending time with friends and enjoying entertainment, for middle-aged and older adults as well as the unique satisfaction of digital leisure activities, such as those that offer personal enrichment [20]. For middle-aged and older adults, using the internet to participate in online leisure activities is accessible, effective, and has a low cost. Traditional leisure activities that require time, money, and physical effort are increasingly available in an online environment, such as video-chatting with friends and playing games online with friends. Particularly during the pandemic, middle-aged and older adults gain social and emotional support as well as rich content experiences by engaging in these online activities, and this digital leisure participation enhances their well-being [22].

Although the internet can enrich the forms of leisure activities in which older adults participate, thus boosting their happiness, quality of life, and well-being, inappropriate internet use, such as prolonged use of the internet, poses a threat to the well-being of middle-aged and older adults. Researchers have used different terms, such as “problematic internet use”, “excessive internet use”, “compulsive internet use”, and “internet dependency”, to describe the prolonged use of the internet and the various mental, physical, and social relationship damages that result. One of the most widely used terms by researchers is internet addiction. In short, internet addiction refers to the uncontrolled impulse to use the internet without the effects of addictive substances and is manifested by significant academic, occupational, or social impairment resulting from overuse of the internet [23]. Although Young’s definition of internet addiction indicates that the internet use patterns and behavioral performance of many older Chinese people today meet his criteria, we suggest that it is inappropriate and risky to use the term internet addiction to describe internet use among middle-aged and older adults. We prefer to use excessive internet use to describe this phenomenon. Excessive internet use, which also refers to excessive or poorly controlled preoccupations, urges, or behaviors regarding internet access that lead to impairment or distress, has a connotation similar to that of internet addiction [24]. The problem with the term internet addiction is that addiction should be a strictly pathological diagnosis, which is difficult to identify through social observation or surveys. However, researchers have not yet reached a unified opinion on what level of dependency can be considered addiction or even the clinical criteria for internet addiction [24]. This has been the main challenge in defining and measuring internet addiction as proposed by Young (2004). In addition, the risk of using the term addiction is that we can easily be reduced to a critique of individual traits and patterns of internet use from a God perspective, and this can lead to the occurrence of many abnormal and nonscientific corrective measures for internet addiction, such as the treatment of adolescent internet addiction that has taken place in many parts of China, objectively undermining the physical and mental health of adolescents [25]. The internet has already become a social infrastructure and inevitable destiny of modern people; thus, we should abandon this implied critique of the individual and consider the social implications and consequences of internet use in a broader social context. In this study, we chose to use the term excessive internet use to describe the inappropriate and prolonged use of the internet by middle-aged and older adults.

The study of excessive internet use has attracted scholarly attention since the early emergence of the internet, with sustained and extensive, cross-national, comparative, and panel studies on the concept, measurement, prevalence, and treatment of excessive internet use having been carried out. Nevertheless, these studies focused on adolescents or college students and rarely extended the concept to other age groups. The occurrence of excessive internet use has been tacitly assumed to be a phenomenon that exists only at specific life stages, especially in adolescence. However, as the internet has become a social infrastructure and a shared way of life for people in the digital era [26], different groups of people, including middle-aged and elderly individuals, are involved in it to varying degrees. From the perspectives of innovation diffusion, technology adoption, or use and gratification, many researchers have studied middle-aged and older adults’ use of various types of digital media, their motivations, and barriers as they integrate into the digital era [9,10,27]. Little attention has been given to the individual and societal consequences of digital media use by middle-aged and older adults, especially the consequences of inappropriate media use. We suggest that it is crucial to be concerned about the inappropriate use of the internet (e.g., internet overuse) by middle-aged and older adults from both individual and societal perspectives, as these inappropriate internet uses tend to expand in the context of accelerating global aging and are directly related to the public health and well-being of middle-aged and older adults.

The emergence of this phenomenon is an inevitable consequence of the intertwining of an aging society and the rapid development of ICT. It seems to contradict our perception that the middle-aged and the elderly tend to be more mature, rational, and mentally sound than teenagers and are often perceived to have stronger resistance to new things such as the internet [7]. However, the heavy use of the internet by an increasing number of middle-aged and older adults makes this commonly held perception seem inaccurate. How can this phenomenon be explained? A widely spread and shared view is that a lack of care in reality leads to a prolonged and intensive search for alternative emotional satisfaction on the internet, and the lonely state of relatively old adults leads to excessive internet use among them. However, loneliness as a psychological state in individuals has been found in previous studies to be a mediating factor rather than an essential cause of misbehavior, such as alcohol and high-risk medication abuse, in general [28]. To argue that middle-aged and older adults use the internet intensely because they are driven by loneliness is to take the gap between external environmental factors and the desired state of middle-aged and older adults as the cause of their excessive internet use, which is still essentially viewing their internet use as a passive acceptance process. The subjective and preconceived attribution of excessive internet use among the middle-aged and the elderly to loneliness may seem to be in line with common sense, but it makes us ignore many deeper and more essential factors that lead to excessive internet use among them and prevents us from understanding the problem correctly, and the related interventions certainly produce a greater bias. Is the internet use of middle-aged and older adults so passive? Many previous studies have answered this question negatively. Older individuals have favorable intentions when using the internet. The richness of internet content and its entertaining features make it a contributing factor to sensation seeking among middle-aged and older adults, and the use of the internet increases their happiness [29]. Therefore, does this active sensation seeking further contribute to excessive internet use? To what extent can loneliness be used to explain middle-aged and older adults’ excessive internet use, and how does it work? Our study addresses these questions.

### 2.2. Sensation Seeking and Excessive Internet Use

Sensation seeking has proven to be a valid explanatory variable in studies of various addictive phenomena, such as gambling, alcohol abuse, and drug abuse [30]. It is often discussed as a risk factor for hazardous and maladaptive behavior, particularly during the transition into adulthood [31]. It refers to personality traits that are associated with a variety of behavioral problems as individuals move away from monotony and seek diverse, novel sensory experiences, maintaining relative stability across the life course. Some studies found that sensation seeking changes over time, with sensation-seeking traits remaining relatively stable in high and low sensation seekers while increasing with age in moderate sensation seekers [32]. Previous research has rarely examined middle-aged and older adults’ internet use from a sensation-seeking perspective, often assuming that they are naturally resistant to new things such as the internet; thus, they have focused more on factors related to their internet use from a technology adoption perspective. Sensation seeking is normally distributed across age groups, and its relative stability allows individuals to maintain or even increase their sensation-seeking traits into adulthood or old age, contributing to their risk of excessive internet use. Thus, we hypothesize the following:

**H1.**  Sensation seeking positively predicts excessive internet use in middle-aged and older adults.

Sensation seeking contains four subdimensions: thrill and adventure seeking, experience seeking, disinhibition, and boredom susceptibility. Studies have confirmed that all four dimensions have a positive effect on excessive internet use [33]. People with high levels of sensation seeking tend to tire more easily of the repetitive and predictable experiences of everyday life and are more eager to seek new and diverse experiences. The internet can be used to take risks and explore interesting and novel phenomena. The internet is one of the best ways to satisfy feelings of excitement [34]. Thus, we hypothesize the following:

**H1a.**  Thrill and adventure seeking positively predicts excessive internet use among middle-aged and older adults.

**H1b.**  Experience seeking positively predicts excessive internet use among middle-aged and older adults.

**H1c.**  Disinhibition positively predicts excessive internet use among middle-aged and older adults.

**H1d.**  Boredom susceptibility positively predicts excessive internet use among middle-aged and older adults.

Although most studies have found that all four subdimensions of sensation seeking positively predict excessive internet use [35], the relative influence of these four dimensions varies across different types of internet use and subpopulations, and there is no consensus. In other words, we want to know which subdimension is the most important risk factor for excessive internet use by middle-aged and older adults among the four subdimensions of sensation seeking. Several studies have found that disinhibition is most associated with excessive internet use and is the main risk factor for excessive adolescent internet use [33,36]. This may be because adolescents are in a rebellious phase of their development and seek to establish personal identity by breaking social norms, even reaching the edge of being anti-establishment [37]. For middle-aged and older adults, however, it seems to be different. Middle-aged and older adults, as socially experienced, sound, and mentally mature individuals, tend to be aware of and comfortable with various social norms and rarely exhibit social rebelliousness and are usually defenders of social norms [38]. In this situation, disinhibition does not seem to explain their dependency on the online world. We propose that experience seeking may better explain their internet use than disinhibition. As digital immigrants, middle-aged and older adults have the curiosity to experience new things such as the internet as a way to experience new things they cannot access in their daily lives and to gain novel experiences. Thus, we hypothesize the following:

**H2.**  Experience seeking has the greatest impact on middle-aged and older adults’ excessive internet use among the four subdimensions of sensation seeking.

### 2.3. The Mediating Role of Loneliness

Loneliness can be defined as the reaction to a lack of necessary social relations or, despite social relations, having a lack of warmth, intimacy, and emotions. It is a state of dissatisfaction resulting from a discrepancy between an individual’s desired and actual social relationships [39]. Research has provided evidence that loneliness and social anxiety are positively associated with problematic internet use. Internet-addicted individuals tend to have higher levels of loneliness and frequent urges to go online for emotional support [40]. The use of the internet is effective in reducing loneliness in middle-aged and older adults; the frequency of internet use is negatively associated with loneliness, especially during the pandemic, and greater internet use for online communication is effective in alleviating their loneliness [41,42]. Internet use among middle-aged and older adults promotes their subjective well-being through the mediation of loneliness [43]. Thus, we hypothesize the following:

**H3.**  Loneliness positively predicts excessive internet use in middle-aged and older adults.

Although many previous studies have found loneliness to be a significant predictor of excessive internet use, loneliness often mediates the effects of various individual, social, and environmental factors on excessive internet use but is not the essential cause of the problem. For example, a study found that the quality of social relationships is a strong predictor of excessive internet use and that loneliness partially mediates the association between the quality of social relationships and excessive internet use [44]. Furthermore, the ability to initiate social relationships and loneliness sequentially mediate the relationship between low self-esteem and excessive internet use [45]. The adaptation of social relationships and problematic internet use, such as excessive internet use, are also mediated by loneliness [46]. However, these studies have invariably been conducted in youth groups, and direct evidence of the mediating role of loneliness in middle-aged and older adults’ excessive internet use has not been available. Due to differences in life stages, loneliness in youth tends to result from social integration problems in the early socialization stages, whereas loneliness in older adults results from the weakening of preexisting social ties and the absence of social care in the later stages of life. The essential difference between these two makes it necessary to study the mediating role of loneliness in older adults’ excessive internet use. We propose that loneliness in middle-aged and older adults is stimulated by unfulfilled sensation seeking in life and exacerbated by diminishing socialization in later life. The use of the internet, on the other hand, is both easy and attractive. Unfortunately, repeated use of the internet to alleviate loneliness may lead to excessive internet use or the development of dependence. In addition, previous studies have tended to examine sensation seeking as a holistic concept, so the relationship between its four subdimensions and loneliness lacks necessary research. We believe that a further test of mediating effects could help us to better understand and intervene in middle-aged and older adults’ excessive internet use. Thus, we hypothesize the following:

**H4a.**  Loneliness mediates the relationship between thrill and adventure seeking and excessive internet use.

**H4b.**  Loneliness mediates the relationship between experience seeking and excessive internet use.

**H4c.**  Loneliness mediates the relationship between disinhibition and excessive internet use.

**H4d.**  Loneliness mediates the relationship between boredom susceptibility and excessive internet use.

## 3. Method

### 3.1. Samples

We conducted an online panel survey using the Tencent database in October 2021. Online panels can be more precisely targeted to a study population and improve the response rate of a survey. Therefore, they have been increasingly used as a popular and efficient method for data collection [47]. The target population in this study was people aged 50 or older because 50 is the legal retirement age for female workers in China, and 60 is the legal retirement age for men. Older adults in this age group are facing or are already in retirement, and their life status will change significantly during this period. Tencent is the largest internet company in China, with over 1.26 billion users. We used Tencent’s database to push a questionnaire to users based on the age range we selected. Once users received the pushed questionnaire, they could choose whether or not to open the questionnaire link according to their own wishes. We also informed users of the purpose and target audience of the survey on the first page of the questionnaire, and users could decide whether to fill out the questionnaire based on their own interests with informed consent. In the process of the survey, respondents could exit the questionnaire and end the survey at any time. The response rate of the questionnaire was 60%, *N* = 446. The demographic characteristics of the sample are shown in Table 1.

### 3.2. Measurement

#### 3.2.1. Excessive Internet Use and Related Problems

The dependent variable was excessive internet use. Many researchers have given criteria for what intensity of internet use can be considered excessive based on the amount of time an individual spends online. For example, Young (2014) suggests that individuals who regularly use the internet for more than 40 h per week are considered to be internet addicts. However, we believe that using the length of internet use as a criterion, although easy to apply, is not adequate. The length of time spent on the internet and the purpose of internet access differ for individuals due to different life and work demands, so using the time spent on the internet as a criterion for judging internet dependence is not sufficient. Especially during the pandemic, school, work, and interpersonal communication were conducted online to a large extent, which substantially increased the time spent online [11]. Therefore, for the measurement of excessive internet use, we were more concerned with the effects of prolonged internet use on the physiology and psychology of older adults and their reactions after internet withdrawal.

Considering the cultural proximity between mainland China and Taiwan, we used the Core Symptoms of Internet Addiction subscale of the Revised Chinese Internet Addiction Scale (CIAS-R) to measure excessive internet use. It is a self-report questionnaire with a four-point Likert scale ranging from 1—*Does not match my experience at all* to 4—*Definitely matches my experience*. The total score represents the degree of excessive internet use; the higher the total score is, the higher the degree of excessive internet use. Considering the ethnic, historical, and sociocultural consistency between mainland China and Taiwan, we believed that using this scale could more accurately measure the excessive internet use status of the Chinese public. The measured reliability coefficient was α = 0.944.

In addition, we measured related problems caused by excessive internet use with the subscale Related Problems of Internet Addiction of Chen’s (2013) CIAS-R scale. The problems related to excessive internet use mainly refer to interpersonal and health problems, such as alienation and impairment of interpersonal relationships, reduction in leisure activity time in reality, and an increase in physical discomfort brought about by prolonged internet use, which reflect the specific consequences of inappropriate internet use behaviors. The measured reliability coefficient was α = 0.842.

#### 3.2.2. Sensation Seeking

Because the expressions and judgments of sensation seeking vary greatly across cultures, we used the Brief Sensation Seeking Scale-Chinese (BSSS-C) as our scale for assessing sensation seeking in middle-aged and older Chinese adults to avoid cultural bias. The scale consists of 8 questions, with 2 questions measuring each of the 4 subdimensions of the respondent’s sensation-seeking traits. A five-point Likert scale is used, with answers ranging from 1—*strongly disagree* to 5—*strongly agree*, with higher scores indicating a stronger degree of sensation seeking. Its reliability has been well validated in empirical studies in China [48]. In our study, the reliability of our measurement using this scale was α = 0.837.

#### 3.2.3. Loneliness

We used the UCLA-3 Loneliness Scale to measure loneliness. The scale measures the gap between an individual’s expected social relationships and their actual social situation using a four-point Likert scale in which nine items are reverse scored, with higher total scores representing greater loneliness for the respondent. In China, this scale has been confirmed by factor analysis to be a good tool for the measurement of loneliness [49]. In this study, the measurement reliability of the scale was α = 0.784.

#### 3.2.4. Demographic Variables and Functions of Internet Usage

In addition to the above variables, we also asked respondents about demographic attributes such as gender, age, education level, income, occupation, marital status, child companionship, and health status. In addition, we asked respondents about the time they spend on the internet and the main functions they use it for. For the functions of internet use, we listed nine common functions of the internet. Respondents were asked how often they use each function, ranging from 1—*never use* to 5—*always use*. After exploratory factor analysis (KMO = 0.812, *p* < 0.001), the functions of internet use were divided into 3 categories: leisure activities, life services, and work and study (Table 2). We used the scores of each factor for further analysis.

## 4. Findings

### 4.1. Common Method Bias Test

Since the items in our questionnaire were all self-reported, there was potential for common method bias causing artificial covariate relationships that interfere with the authenticity of the findings, so the test for common method bias was conducted first. In the survey procedures, we reduced common method bias by emphasizing the anonymity of the questionnaires and using methods such as reverse scoring. On the other hand, the collected data were examined by the Harmen one-way test to examine the extent to which the correlation of variables was due to common method bias, and it was found that 15 factors in the unrotated factor analysis had eigenvalues greater than 1, and the first factor explained only 19.7% of the variance, which was below the 40% determination criterion, indicating that there was no serious common method bias.

### 4.2. Internet Use among Middle-Aged and Elderly Adults

Table 2 demonstrates the use of the common internet functions listed by middle-aged and older adults and the results of the factor analysis. As seen from the table, the highest level of internet use was for financial payments (M = 4.11) and online social interaction (m = 4.05). This is consistent with previous researchers’ findings that one of the main drivers for Chinese older adults to adapt to the internet is to socialize, and the need for socialization increases rather than decreases with age [50]. Regarding the use of the internet for entertainment, respondents reported a high level of use of audio-visual entertainment, such as various video apps (M = 3.74). The least used function by participants was game playing (M = 2.77). This reflects the group characteristics of middle-aged and older adults, who are more likely to consume entertainment content and less likely to engage in online content with exciting and adventurous features and who use the internet for leisure activities.

Table 3 shows the correlation coefficient of the variables we measured. Respondents reported a high level of excessive internet use (M = 2.65, ranging from 1 to 4), which is consistent with our observations and suggests that excessive internet use among middle-aged and older adults is an objective fact that cannot be overlooked. Nevertheless, respondents’ self-reported loneliness was not strong (M = 1.87), suggesting that we perhaps overestimated the impact of loneliness on their excessive internet use. On all four subdimensions of sensation seeking, respondents reported moderate-to-high levels of sensation seeking intensity. Therefore, compared to loneliness, sensation seeking may be more closely related to excessive internet use.

In terms of internet functions used, life services and work and study, as well as leisure activities, were positively correlated with excessive internet use, and leisure activities had the highest correlation coefficient with excessive internet use. The entertainment function of the internet tends to encourage greater dependency among users, and middle-aged and older individuals are no exception. In terms of loneliness, life services and work and study were negatively related to loneliness, while leisure was positively related to loneliness, implying that middle-aged and older adults’ use of the internet for leisure activities may increase their loneliness, which, in turn, causes excessive use of the internet.

### 4.3. Regression Analysis

We conducted multiple regression analyses with excessive internet use and loneliness as dependent variables, and the results of the regression are shown in Table 4 (Model 1 to Model 3).

After controlling for the demographic variables, we found that the regression coefficient of sensation seeking on excessive internet use was β = 0.413 (*p* < 0.001) when we combined the four subdimensions of sensation seeking; thus, hypothesis 1, that the higher the sensation seeking is, the more intense the excessive internet use, was accepted. As Model 1 shows in Table 4, the direct effects of all four dimensions of sensation seeking on excessive internet use were significant (*p* < 0.01), and experience seeking (β = 0.255), disinhibition (β = 0.224), and boredom susceptibility (β = 0.137) positively predicted excessive internet use by middle-aged and older adults, while thrill and adventure seeking negatively predicted middle-aged and older adults’ excessive internet use (β = −0.173). Therefore, hypothesis 1a was rejected, and hypotheses 1b, 1c, and 1d were accepted. Hypothesis 2 was supported because the standardized regression coefficient of experience seeking (β = 0.276) was larger than any other dimension and was the most influential risk factor for excessive internet use among middle-aged and elderly individuals.

Subsequently, we conducted a multiple regression analysis with loneliness as the dependent variable (Table 4, Model 2). The subdimensions thrill and adventure seeking were still negative predictors of loneliness (β = −0.156), experience seeking negatively predicted loneliness (β = −0.171), disinhibition positively predicted loneliness (β = 0.35), and the effect of boredom susceptibility on loneliness was not significant (*p* > 0.05). In terms of the functional use of the internet, life services, as well as work and study, were negatively associated with loneliness (*p* < 0.05), while using the internet for leisure activities was positively associated with stronger feelings of loneliness (β = 0.139, *p* < 0.01).

Later, after adding loneliness into the model (Table 4, Model 3), loneliness positively predicted the excessive internet use of middle-aged and older adults (β = 0.121, *p* < 0.01) after controlling for the covariates as well as the four dimensions of sensation seeking; thus, hypothesis 3 was accepted. Using the internet for leisure activities increased the loneliness of middle-aged and older adults, which, in turn, promoted their excessive use of the internet (β = 0.142, *p* < 0.01).

Among the four subdimensions of sensation seeking, contrary to our hypothesis, thrill and adventure seeking had a stable, significant negative effect on both loneliness and excessive internet use. That is, the higher the respondents’ thrill and adventure seeking, the lower their feelings of loneliness and excessive internet use. This finding is contrary to almost all previous studies conducted on adolescents. Why? We propose that, unlike adolescents, who are addicted to various stimulating content on the internet, middle-aged and older adults use the internet more to relieve loneliness and rarely participate in stimulating activities such as online games. Therefore, although thrill and adventure seeking maintains relative stability across life, it has different expressions at different life stages. For middle-aged and older adults, this adventure personality trait is more about physical activities rather than screen activities [51], and their adventure traits are more likely to manifest in real-life activities such as hiking and rafting. In turn, the more energy they devote to such real-life activities, the less likely they are to perceive loneliness and indulge on the internet.

Further regression analysis with related problems as the dependent variable (Table 4, Model 4) showed that excessive internet use significantly alters the physical and social conditions of middle-aged and older adults, with higher intensity of use implying greater health stress and more distant real-life interpersonal relationships (β = 0.739, *p* < 0.001). The potential negative impact of excessive internet use on middle-aged and older adults is a cause for concern.

### 4.4. Tests of Mediation Effects of Loneliness

A further mediation effects test of loneliness was conducted to test whether loneliness mediates the effect of sensation seeking on excessive internet use. The mediation effect is valid if the three variables are correlated with each other. As seen from the correlation matrix in Table 2, the correlations between excessive internet use, loneliness, and the four subdimensions of sensation seeking were all significant, so a test of the mediating effect could be conducted.

We used the PROCESS macro for the test of mediating effects (Table 5). After standardization of variables and controlling covariates such as gender, age, education level, retirement status, residence, and income per month, the mediation effect test of loneliness showed that there was no significance between sensation seeking and excessive internet use (95% CI = (−0.0239, 0.0198)), and only a significant direct effect existed (t = 8.39, *p* < 0.001). Considering that thrill and adventure seeking acts in an opposite direction to the other three subdimensions of sensation seeking, we suggest that the insignificance of this mediating effect may have been confounded by this opposing force and, thus, did not reach a statistically significant level.

A further test of the four subdimensions of sensation seeking revealed varying results. Loneliness partly mediated the relationship between experience seeking (95% CI = (−0.0526, −0.0121)), disinhibition (95% CI = (0.0006, 0.0228)), boredom susceptibility (95% CI = (0.0027, 0.0459)), and excessive internet use, but it did not mediate the association between thrill and adventure seeking and excessive internet use (95% CI = (−0.0194, 0.0014)). Since the direct effects of experience seeking (β = 0.37), disinhibition (β = 0.32), and boredom susceptibility (β = 0.18) on excessive internet use were all significant (*p* < 0.001), loneliness as a mediator partially mediated the association between experience seeking, disinhibition, boredom susceptibility, and excessive internet use in middle-aged and older adults. Therefore, hypotheses 4b, 4c, and 4d were verified, and hypothesis 4a was rejected. Conclusively, the results of hypothesis testing in this study are shown in Table 6. Except for hypotheses H1a and H4a, which were rejected, the rest of the study hypotheses were verified.

## 5. Discussion

### 5.1. Key Findings and Implications

This study focused on the phenomenon of excessive internet use by middle-aged and older adults in mainland China and has two main findings. First, middle-aged and older adults show both the general characteristics of the problem and the specificity of belonging to the age group in terms of excessive internet use. The generality lies in the fact that we found personality-driven proactivity in middle-aged and older adults’ internet use and that sensation seeking as a genetic personality factor steadily influences internet use across different age groups with no exception for middle-aged and older adults. The specificity lies in the fact that sensation seeking has a more complex effect on middle-aged and older adults than on other groups, such as adolescents, which previous studies revealed. The influence of the subdimensions of sensation seeking on excessive internet use changes accordingly with age, and some risk factors are, instead, transformed into inhibitors of excessive internet use due to the group characteristics of middle-aged and older adults. That is, experience seeking, disinhibition, and boredom susceptibility positively predict excessive internet use in middle-aged and older adults. However, thrill and adventure seeking were found to have a protective effect on excessive internet use in middle-aged and older adults. Among the four dimensions of sensation seeking, unlike the strongest explanatory power of disinhibition found in previous studies, middle-aged and older adults are most strongly influenced by experience seeking. This indicates that adolescents are more likely to seek a virtual environment for individual emotional release from the real world and use the internet as an alternative space to real life, which leads to excessive internet use. Middle-aged and older adults are more likely to use the internet as an extension of real life to gain various experiences that are not accessible in real life, which leads to excessive use of the internet.

Second, loneliness is an effective but relatively limited explanation for middle-aged and older adults’ excessive internet use. Our research confirms the mediating role of loneliness, which is an important mediator for middle-aged and older people seeking novel experiences on the internet that are not available in real life. As middle-aged and older people are at a relatively late stage in their lives, they experience varying degrees of a lack of social attention, family care, and child companionship. As the real world fails to meet their emotional needs, they turn to the online world for experiential satisfaction and emotional comfort, which increases the risk of excessive internet use. With further aging in the future, it is expected that this problem will become more apparent in a wider range of areas and groups. Despite this, however, the effect of loneliness is relatively limited, as its regression coefficient was significant but not high, even lower than for each subdimension of sensation seeking. This indicates that loneliness is not an essential factor of excessive internet use among older adults but just a psychological mediator. It is completely untenable to attribute the excessive internet use of middle-aged and older adults exclusively to their loneliness. Intervening in the problem of excessive internet use among middle-aged and older adults from the perspective of reducing their loneliness would produce a directional bias. We believe that meeting their sensation-seeking needs is key to reducing high-intensity internet use and mitigating health risks in middle-aged and older adults, not reducing loneliness. Therefore, to reduce the dependence of middle-aged and older adults on the internet and guide them to form healthy internet habits, it is necessary to satisfy their sensation-seeking needs by replacing the emotional satisfaction they receive from the internet. We suggest leading middle-aged and older adults to participate more in real-world leisure activities, such as outdoor trips, mountain climbing, and other physical sports, to spend their leisure time and energy on the one hand and enhance their overall physical quality on the other hand so they can satisfy their needs for thrill and adventure seeking and reduce internet use.

### 5.2. Theoretical Contribution

The theoretical contribution of this paper lies in its research perspective and findings, which provide a timely and necessary complement to previous works on excessive internet use by identifying specific differences that shape excessive internet use among middle-aged and older adults and provide empirical evidence for effective health interventions. In terms of research perspective, this study focused on the potential consequence of middle-aged and older adults’ internet use rather than on their adoption and use pattern. From a consequence perspective, we extended the study of excessive internet use to the aged population and confirmed that, in addition to the youth group, which was the focus of previous studies, the relatively old population also has a susceptibility to excessive internet use. This suggests that our stereotype of relatively old adults as laggards in the digital media era is unreliable and that they do not have special resistance to the negative effects of the internet, such as excessive internet use. Excessive internet use is not only a problem that is widespread across space in different countries and cultures, as previous studies have confirmed, but also an ephemeral problem that occurs across generations.

There is no denying that the internet has brought tremendous convenience and rich experiences to seniors in the relatively late stages of their life course. Many studies have demonstrated a positive association between internet use and subjective well-being among older adults, with leisure being the most strongly associated with subjective well-being [52,53]. Through strategic use, the internet can be used to support the personal and social resources that they have. For older adults, the internet can even provide a quality-of-life technology that maintains or enhances functioning in later life [54]. The role of the internet in facilitating the health and well-being of middle-aged and older adults has been highlighted, especially during the pandemic. Reduced opportunities for recreation and exercise in the real world have made the internet an alternative option for engaging in physical activity and social interaction, and middle-aged and older adults have used the internet to access online recreational resources and follow pre-recorded classes to exercise, and to access information and emotional support through online communities, which is beneficial for their mental health and counters isolation [22,55]. Our purpose is not to deny the importance of the internet in promoting the well-being of middle-aged and older adults but only to caution that such use should be moderate, healthy, and bounded. Once the boundaries of moderation are exceeded, the negative effects of the internet become just as dramatically detrimental to the health and well-being of middle-aged and older adults, with many unintended consequences.

Because middle-aged and older adults are at a relatively late stage in their lives, excessive internet use at this stage places a heavy burden on their health and well-being, as our study revealed. With the further increase in internet coverage and the penetration of the internet into social life, this problem has the potential to become a public health issue of concern.

We believe that, in an era when the internet has become one of the common infrastructures of the global community and has comprehensively penetrated the daily lives of people worldwide, it is essential to profoundly reflect on the social significance of the internet. The internet as an infrastructure implies that the various individual-level and social-level effects of the internet, whether positive or negative, will become a common destiny for the public in the digital age. For modern people, this differs only in the degree of involvement and forms of expression, with little possibility of escape from digital space. Star and Bowker (1996) proposed that an infrastructure is not a completely independent, objective reality or a set of transcendent systems but a relationship, i.e., the way individuals use it [56]. When individuals can make effective use of an infrastructure, it is a facilitating infrastructure; conversely, when individuals are unable to make full use of an infrastructure or when the use of an infrastructure results in undesired effects, the infrastructure is a barrier [57]. From this relational framework, any infrastructure is of a social rather than purely technical nature, and the internet is no exception. The social nature of the internet means that, as an infrastructure that cannot be avoided, the differential use of the internet by different groups of people has the potential to generate unintended consequences. Our society is accustomed to viewing middle-aged and older adults from the perspective of media dominance. The prevailing stereotypes of older people and an artificial definition of them as lonely and technologically resistant are misleading, so we have spared no effort to market the new digital technologies and internet features to older people to integrate them into the digital age and help them realize “active aging” [58]. When we shift our perspective to that of elderly individuals, as this study reveals, we wrongly presuppose the mentality of elderly individuals. Rather than the stereotypical image of a stubborn resister to new technologies, middle-aged and older adults have an intense and active need to use the internet to pursue a variety of emotional experiences that cannot be satisfied otherwise, even developing excessive use and dependency on the internet. The excessive use of the internet for leisure also threatens the socialization process of middle-aged and older people in the later stages of their lives and their well-being, which objectively leads to “passive aging”. Therefore, any infrastructure is social in nature, and the internet as digital infrastructure in a universal sense can also be a social barrier to inclusion for vulnerable groups such as relatively old individuals. Whether it enhances or diminishes well-being depends on how and why people use it, as well as who uses it [59]. Our society, however, envisions the existence of a digital utopia where older people can enjoy the convenience and richness of life brought about by the development of digital media technology with little risk of being affected by the negative effects of digital technology. Therefore, all older people need to embrace the digital utopia, which will enhance their well-being as well as their quality of life and enable them to achieve social equality in the digital age. There is no doubt that this view is short-sighted and harmful; ignoring the premise that middle-aged and older people are a vulnerable group in terms of media literacy makes such undifferentiated digital inclusion likely to have unintended and pernicious consequences. Therefore, as a foundation of modern social life, researchers and policymakers need to fully understand the complexity of the internet and focus on its social implications for different groups. Going digital alone is insufficient to help middle-aged and older adults obtain a better life. Coupling online and offline strategies is invaluable in addressing the challenges middle-aged and older adults face [60].

### 5.3. Limitations

There are some limitations in this study. First, due to the lack of a unified definition and measurement of excessive internet use, the excessive internet use scale we used does not provide a reference value to determine whether a respondent can be considered internet addicted but rather a relative measure, which may affect our understanding of the overall situation of excessive internet use among middle-aged and older adults. Second, our study focused only on the effect of sensation seeking on middle-aged and older adults’ excessive internet use to demonstrate that they have personality factors that lead to excessive internet use without taking various other factors, such as the external environment, into account, and future studies could study this issue from a richer perspective. Finally, due to the limitations of the survey platform, the sample we obtained was predominantly middle-aged people aged 50–55 years; thus, the study population for this study was middle-aged and a small percentage of elderly people. Future studies could be conducted in older age groups to better confirm the salience of this social issue.

## Figures and Tables

**Table 1:** Descriptive statistical results of samples (*N* = 446).

Properties	Category	n	Percentage (%)
Gender	Male	218	48.9
	Female	228	51.1
Age	50–55	322	72.2
	56–60	88	19.7
	61–65	20	4.5
	Over 65	16	3.6
Residence	Rural	122	27.4
	Cities and towns	324	72.6
Child Companionship	Yes	246	55.2
	No	200	44.8
Retirement Status	Retired	160	35.9
	Unretired	286	64.1
Occupation	No work	32	7.2
	Farmers	56	12.6
	Public officials	114	25.6
	Corporate staff	102	22.9
	Workers	72	16.1
	Self-employed	40	9.0
	Other	30	6.7
Income Per Month	<2000 RMB	80	17.9
	2000–4000 RMB	120	26.9
	4001–6000 RMB	104	23.3
	6001–8000 RMB	54	12.1
	>8000 RMB	88	19.7
Education Level	Elementary school or less	16	3.6
	Junior high school	82	18.4
	High school/technical school	104	23.3
	College	116	26.0
	Undergraduate	116	26.0
	Master’s degree or more	12	2.7
Health Status	Very poor	2	0.4
	Poor	54	12.1
	Good	292	65.5
	Very good	98	22

**Table 2:** Mean, standard deviation, and factor analysis of functions of internet use by middle-aged and older adults.

Functions of Internet Use	Examples	Mean	SD	Factor 1 Life Services	Factor 2 Work and Study	Factor 3 Leisure Activities
Financial payments	Internet banking, Alipay	4.11	0.864	0.838		
Online shopping	Group buying, Taobao	3.84	0.822	0.788		
Online social	Instant messaging, social media sites	4.05	0.762	0.696		
Daily service	Room reservation, taxi, health code	3.5	0.994	0.617		
Working	Document processing, online meetings	3.32	1.163		0.860	
Learning	Online education, learning skills	3.52	0.933		0.723	
Get news and information	Various news apps	3.9	0.826		0.622	
Audio/video entertainment	Movies, short videos	3.74	0.927			0.839
Games	Single-player games, online games	2.77	1.184			0.741

Note: *N* = 446. Maximum variance rotation factor analysis of functions of internet use.

**Table 3:** Mean, standard deviation, and correlation among variables.

Variables	M	SD	1	2	3	4	5	6	7	8	9	10
1. Life services	-	-	-									
2. Work and study	-	-	0.000	-								
3. Leisure activities	-	-	0.000	0.000	-							
4. Thrill and adventure seeking	3.14	0.90	0.191 **	0.306 **	0.096 *	-						
5. Experience seeking	3.61	0.77	0.308 **	0.344 **	0.089	0.546 **	-					
6. Disinhibition	2.46	0.91	0.064	0.196 **	0.146 **	0.647 **	0.381 **	-				
7. Boredom susceptibility	3.16	0.88	0.085	0.147 **	0.155 **	0.403 **	0.327 **	0.437 **	-			
8. Loneliness	1.87	0.41	−0.169 **	−0.082	0.122 **	0.071 *	0.185 **	0.148 **	0.076 **	-		
9. Excessive internet use	2.65	0.56	0.111 *	0.106 *	0.263 **	0.241 **	0.330 **	0.384 **	0.324 **	0.152 **	-	
10. Related problems	2.78	0.57	0.044	0.112 *	0.227 **	0.156 **	0.212 **	0.320 **	0.235 **	0.208 **	0.783 **	-

Note: *N* = 446, * *p* < 0.05, ** *p* < 0.01.

**Table 4:** Multiple regression analysis.

Independent Variable	Model 1			Model 2			Model 3			Model 4		
	Excessive Internet Use			Loneliness			Excessive Internet Use			Related Problems		
	B	SE	β	B	SE	β	B	SE	β	B	SE	β
Constant	1.762	0.180	-	2.306	0.140	-	1.368	0.228	-	1.125	0.173	-
Male	−0.084	0.053	−0.076	0.049	0.042	0.060	−0.093	0.053	−0.084	−0.012	0.039	−0.010
Age	0.055	0.035	0.073	−0.022	0.027	−0.039	0.059	0.035	0.078	−0.013	0.025	−0.017
Income per month	−0.042	0.024	−0.104	−0.035	0.018	−0.117	−0.036	0.024	−0.089	−0.008	0.017	−0.018
Education level	−0.004	0.026	−0.010	0.071 ***	0.021	0.213 ***	−0.017	0.027	−0.036	−0.060 *	0.019	−0.128 *
Child companionship	0.075	0.047	0.067	−0.019	0.036	−0.023	0.079	0.046	0.070	0.023	0.034	0.020
Health status	−0.049	0.041	−0.053	−0.173 ***	0.032	−0.252 ***	−0.020	0.042	−0.021	−0.064 *	0.031	−0.067 *
Retirement status	0.029	0.060	0.025	0.050	0.047	0.059	0.020	0.060	0.017	−0.043	0.043	−0.036
Life services	0.038	0.025	0.069	−0.053 **	0.019	−0.129 **	0.047	0.025	0.085	0.008	0.018	0.014
Work and study	0.022	0.026	0.040	−0.044 *	0.021	−0.108 *	0.030	0.026	0.054	0.057 **	0.019	0.100 **
Leisure activities	0.089 ***	0.024	0.160 ***	0.057 **	0.019	0.139 **	0.079 **	0.024	0.142 **	0.027	0.018	0.047
Thrill and adventure seeking	−0.106 **	0.037	−0.173 **	−0.070 *	0.029	−0.156 *	−0.094 *	0.037	−0.154 *	−0.038	0.027	−0.060
Experience seeking	0.184 ***	0.038	0.255 ***	−0.091 **	0.030	−0.171 **	0.200 ***	0.038	0.276 ***	−0.012	0.029	−0.016
Disinhibition	0.148 ***	0.035	0.224 ***	0.157 ***	0.027	0.350 ***	0.171 ***	0.036	0.269 ***	0.039	0.027	0.062
Boredom susceptibility	0.087 **	0.030	0.137 **	0.034	0.023	0.073	0.081 **	0.030	0.128 **	−0.005	0.022	−0.007
Loneliness							0.166 **	0.061	0.121 **	0.095 *	0.045	0.068 *
Excessive internet use										0.76 ***	0.035	0.739 ***
*R^2^*	0.295			0.206			0.308			0.655		
*F*	12.9			7.99			12.735			50.867		
*p*	0.000			0.000			0.000			0.000		

Note: *N* = 446; gender (0 = female, 1 = male), child companionship (0 = no, 1 = yes), and retirement status (0 = no, 1 = yes) are dummy variables. * *p* < 0.05, ** *p* < 0.01, *** *p* < 0.001.

**Table 5:** Mediating effect of loneliness on predicting excessive internet use.

Independent Variable	Direct Effect				Indirect Effects			
	Effect	SE	t	*p*	Effect	SE	LLCI	ULCI
Sensation seeking	0.3024	0.036	8.39	0.000	−0.0005	0.0109	−0.0239	0.0198
Thrill and adventure seeking	0.2846	0.0357	7.967	0.000	−0.0086	0.0053	−0.0194	0.0014
Experience seeking	0.3650	0.0397	9.19	0.000	−0.0277	0.0103	−0.0526	−0.0121
Disinhibition	0.3165	0.034	9.308	0.000	0.0088	0.0053	0.0006	0.0228
Boredom susceptibility	0.1804	0.0278	6.486	0.000	0.0171	0.0097	0.0027	0.0459

Note: The mediating variable was loneliness, and the dependent variable was excessive internet use. Standardized coefficients were estimated with the PROCESS macro in Model 4. The significance of effects was estimated with bootstrapping (5000 samples). The confidence level was 95%. LLCI, ULCI, lower and upper limits of confidence interval.

**Table 6:** Summary of hypothesis testing results.

Research Hypothesis	Results
H1. Sensation seeking positively predicts excessive internet use among middle-aged and older adults.	Verified
H1a. Thrill and adventure seeking positively predicts excessive internet use among middle-aged and older adults.	Rejected
H1b. Experience seeking positively predicts excessive internet use among middle-aged and older adults.	Verified
H1c. Disinhibition positively predicts excessive internet use in middle-aged and older adults.	Verified
H1d. Boredom susceptibility positively predicts excessive internet use in middle-aged and older adults.	Verified
H2. Experience seeking has the greatest impact on middle-aged and older adults’ excessive internet use among the four subdimensions of sensation seeking.	Verified
H3. Loneliness positively predicts excessive internet use in middle-aged and older adults.	Verified
H4a. Loneliness mediates the relationship between thrill and adventure seeking and excessive internet use.	Rejected
H4b. Loneliness mediates the relationship between experience seeking and excessive internet use.	Verified
H4c. Loneliness mediates the relationship between disinhibition and excessive internet use.	Verified
H4d. Loneliness mediates the relationship between boredom susceptibility and excessive internet use.	Verified

## Data Availability

Ethics restrict us on how we can share the data in this paper, which may contain sensitive information regarding the respondents’ physical and mental health condition.
